# Supplementary material for: Contrasting Dynamics of Intracellular and Extracellular Antibiotic Resistance Genes in Response to Nutrient Variations in Aquatic Environments
Source: Antibiotics (Basel). 2024 Aug 28;13(9):817. doi: 10.3390/antibiotics13090817 (PMC11428281; doi:10.3390/antibiotics13090817)
Supplement: Supplementary file 1 [file antibiotics-13-00817-s001.zip › antibiotics-3144881-supplementary.pdf]

Contrasting Dynamics of Intracellular and Extracellular ARGs in Response to Nutrient Variations in Aquatic Environments

Lele Liu [1], Xinyi Zou [1], Yuan Cheng [1], Huihui Li [1], Xueying Zhang [1,*]
and Qingbin Yuan [1,2,*]

1 College of Environmental Science and Engineering, Nanjing Tech University, Nanjing 211816, China; leleliu0221@163.com (L.L.); zou.xinyi@foxmail.com (X.Z.); cy1881579@163.com (Y.C.); 18020117150@163.com (H.L.)
2 State Key Laboratory of Pollution Control and Resource Reuse, School of the Environment, Nanjing University, Nanjing 210023, China
* Correspondence: xueyingzhang@njtech.edu.cn (X.Z.); yuanqb@nju.edu.cn (Q.Y.)

Supporting Information

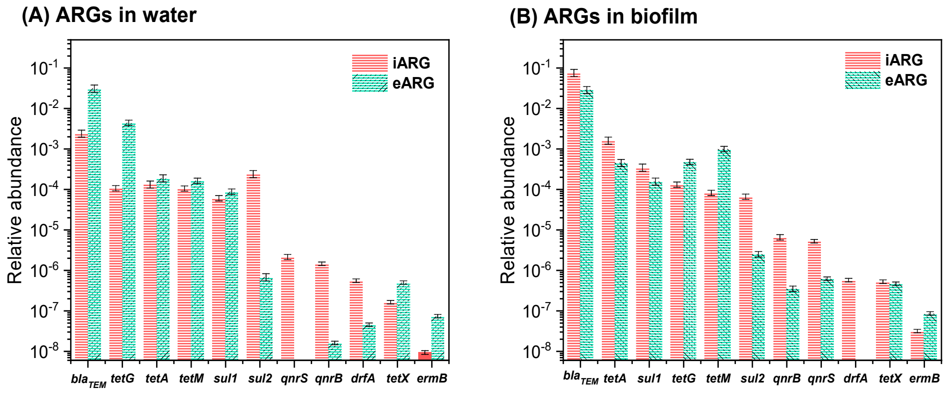

Figure S1. The relative abundance of eleven ARGs in water and biofilms (A, B) in the microcosm experiment.

Table S1. The routine physiochemical and microbial qualities of the water samples

physiochemical parameters	pH	DO (mg/L)	COD_{Mn} (mg/L)	TP (mg/L)	TN (mg/L)	16s rDNA (copies/mL)
values	7.56	6.72	2.7	0.11	1.46	1.43×10^{10}

Table S2. Information of eleven ARGs and 16s rDNA for quantitative PCR operations

gene	Forward primers (5′–3′)	Reverse primers (5′–3′)	Degree of expansion (bp)	Annealing temperatu re (°C)	References
16S rDNA	CCTACGGGAGGCAGCAG	ATTACCGCGGCTGCTGG	193	58	[71]
bla$_{TEM}$	AGCATCTTACGGATGGCATGA	TCCTCCGATCGTTGTCAGAAGT	103	60	[72]
ermB	CCGAACACTAGGGTTGCTC	ATCTGGAACATCTGTGGTATG	139	55	[57]
dfrA	ACGGATCCTGGCTGTTGGTTGG	CGGAATTCACCTTCCGGCTCGA	237	60	[73]
	ACGC	TGTC			
qnrS	GACGTGCTAACTTGCGTG	TGGCATTGTTGGAAACTT	118	54	[74]
qnrB	GGM ATHGAAATTCGCCACTG	TTGCBGYYCGCCAGTCGAA	263	60	[75]
sul1	CACCGGAAACATCGCTGCA	AAGTTCCGCCGCAAGGCT	158	60	[76]
sul2	TCCGGTGGAGGCCGGTATCTGG	CGGGAATGCCATCTGCCTTGAG	191	60	[77]
tetA	GCTACATCCTGCTTGCCTTC	CATAGATCGCCGTGAAGAGG	210	56	[78]
tetM	CATCATAGACACGCCAGGACAT	CGCCATCTTTTGCAGAAATCA	101	60	[79]
	AT				
tetG	GCACGCTGGTTTGGCTACA	TGGCTGTGATTAGTCTCCTTGA	176	56	[76]
tetX	AGCCTTACCAATGGGTGTAAA	TTCTTACCTTGGACATCCCG	278	60	[80]

Table S3. Range of values of relevant parameters of the population dynamics model

parameters	values	parameters	values
r1	0.19–0.21	a	0.0002
r2	0.19–0.21	b	0.0007
M1	50–100	$y\ (1)_0$	4.9×10^{6}
M2	50–100	$y\ (2)_0$	1.6×10^{6}
n	1×10^{10}	$y\ (3)_0$	1×10^{5}
k1	0.0001–0.001	$y\ (4)_0$	2×10^{5}
k2	0.00001–0.0001		
